# Obstetric Outcome and Significance of Labour Induction in a Health Resource Poor Setting

**DOI:** 10.1155/2014/419621

**Published:** 2014-01-20

**Authors:** Osaheni Lucky Lawani, Azubuike Kanario Onyebuchi, Chukwuemeka Anthony Iyoke, Chikezie Nwachukwu Okafo, Leonard Ogbonna Ajah

**Affiliations:** ^1^Department of Obstetrics & Gynecology, Catholic Maternity Hospital, PMB 104, Moniaya, Ogoja, Cross-Rivers State, Nigeria; ^2^Department of Obstetrics & Gynecology, Federal Teaching Hospital, PMB 102, Abakaliki, Ebonyi State, Nigeria; ^3^Department of Obstetrics & Gynecology, University of Nigeria Teaching Hospital, PMB 01129, Enugu State, Enugu 400001, Nigeria; ^4^Department of Obstetrics & Gynecology, Dalhatu Araf Specialist Hospital, PMB 007, Lafia, Nasarawa State, Nigeria

## Abstract

*Objectives*. The aim of this study was to evaluate the methods, indications, outcome of induced labor and its significance in obstetric practice in the study area. *Methods*. This was a retrospective study of cases of induced labor at the Catholic Maternity Hospital in Ogoja, Cross-River State, Nigeria, between January 1, 2002, and December 31, 2011. Data on the sociodemographic characteristics of the parturient, induction methods, indications for induction, outcomes and reasons for failed induction were abstracted from personal case files and the hospital's maternity/delivery register. The data were analyzed with SPSS15.0 window version. *Result*. The induction rate in this study was 11.5%. Induction was successful in 75.9% of cases but failed in 24.1%. Misoprostol was the commonest induction method (78.2%). The commonest indication for induction was postdate pregnancy (45.8%). Failed induction was due to fetal distress, prolonged labor, cephalopelvic disproportion and cord prolapse. The induction-delivery interval was 12 ± 3.6 hours. *Conclusion*. Induction of labor is a common obstetric procedure which is safe and beneficial in well-selected and properly monitored high risk pregnancies where the benefits of early delivery outweigh the risk of continuing the pregnancy.

## 1. Introduction

Induction of labor is the artificial initiation of labor before its spontaneous onset for the purpose of achieving vaginal delivery of the fetoplacental unit [1, 2]. It is a common obstetric procedure which is indicated when the benefits to the mother or fetus outweigh the benefits of continuing the pregnancy [1, 2]. It can involve a complex set of interventions that may defy routines and presents numerous choices and challenges for clinicians and mothers. The rate of induction of labor varies by location and institution but appears to be increasing [1]. In Nigeria a rate of 6.6% was reported in Maiduguri [3]. According to the most current studies in the United States, the rate varies from 9.5 to 33.7 percent of all pregnancies annually [2] One of the most common indication for labor inductions is postterm pregnancy and induction for this indication has been shown to reduce the likelihood of perinatal death [4, 5]. Other indications for induction include premature rupture of membranes especially at term or other situations that require termination of conservative management of high risk pregnancies, potential fetal compromise such as significant fetal growth restriction, nonreassuring fetal surveillance, maternal medical conditions like diabetes, renal disease, significant pulmonary disease, chronic or gestational hypertension, antiphospholipid syndrome, suspected or proven chorioamnionitis, abruptio placentae, and intrauterine fetal death [6–8]. Induction is sometimes performed for “social” or “geographic” reasons, without a medical or obstetric indication [9, 10]. However, there have been few well-designed studies evaluating induction for these indications [11, 12].

Induction when successful results in vaginal delivery but sometimes fails with potential risks of increased rate of operative vaginal delivery, Caesarean birth, excessive uterine activity, abnormal fetal heart rate patterns, uterine rupture, maternal water intoxication, delivery of preterm infant due to incorrect estimation of dates, and possibly cord prolapse [11, 13–17]. Prior to initiation of induction the woman must be assessed for its indications, contraindications to the procedure, gestational age, cervical favorability (Bishop score assessment), and assessment of the pelvis, fetal size, presentation, membrane status (intact or ruptured), and fetal wellbeing; documentation of discussion with the patient including indication for induction and disclosure of risk factors must be undertaken [18]. The state of the cervix is one of the most important predictors of a successful labor induction. In 1964, Bishop described a scoring system based on cervical examination that predicted vaginal delivery in multiparous women [18].

This study was designed to review the induction rate, methods and outcome of induced labor and its significance in obstetric practice in the study area.

## 2. Method and Materials

This was a retrospective study of cases of induced labor at the Catholic Maternity Hospital (CMH) in Ogoja, Cross-River State, South-South, Nigeria, between January 1, 2002, and December 31, 2011. CMH, Ogoja, provide specialized obstetric services to parturient with both complicated and uncomplicated pregnancies. It serves as a referral center and receives patients from Cross-River and other neighboring Nigerian states of Benue and Ebonyi. During the study period a total of 32,584 obstetric patients (booked and unbooked) were managed in the facility with 13,130 deliveries during the same period. Home delivery is a common practice in most Nigerian states with rates as high as 62%. A total of 1510 parturient had induction of labor during the period under review. The maternity and delivery records (patient case notes and maternity/delivery registers) were retrieved from the medical records department. A data entry pro forma was used to abstract necessary data on the sociodemographic characteristics of parturient, induction methods, indications, and outcome of induced labor. This was conducted by resident doctors who were of the rank of registrars and senior registrars. The departmental policy/protocol on induction is that if an induction method fails, a Caesarean section should be undertaken. The main outcomes measured were the proportion of women who had induction of labor, the indications for induction, and the proportion of successful and failed inductions. Statistical analysis was with Statistical Package for the Social Sciences version 15.0 for windows (SPSS; IBM Corporation, Armonk, NY, USA). Conclusions were drawn by means of simple percentages and mean.

## 3. Results

A total of 32,584 obstetric patients were managed during the period under review with 13,130 deliveries recorded, giving a hospital delivery rate of 40.3%; of these 1510 had induction of labor, giving an induction rate of 11.5%. The mean age of the participants was 27.51 ± 8.37 years. The participants had varying levels of education as depicted in Table 1. The vast majority (86.2%) were booked and received antenatal care. The gestational age at booking shows that 80% of parturient booked between 28 and 42 weeks of gestation, indicating that most women in the study environment still have the attitude of booking late in pregnancy, 16.7% booked at 13–27 weeks, 2% at <13 weeks, and 1.3% at >42 weeks. Primigravidae accounted for 28.5% of cases, 7.7% were grand multiparous, while others (63.8%) were Para 1–4 (Table 1). The mean parity of the subjects was 3.6 ± 1.1.

Table 2 shows the methods used for induction of labor to include misoprostol (78.2%), intracervical extra-amniotic Foleys catheter (1.7%), Foleys catheter and amniotomy/oxytocin (9.1%), amniotomy/oxytocin (7.0%), membrane sweep (0.8%), and membrane sweep and amniotomy/oxytocin (3.2%). The indications for induction are also shown in Table 2 where postdate was the commonest indication accounting for 45.8% of inductions.

In all cases where misoprostol was used, 50 micrograms was inserted into the posterior vaginal fornix every 6 hours with only a maximum of 3 doses allowed. Majority (85%) required between 1 and 2 doses, 13% had their labor induced with 3 doses, and 2% had no cervical changes or contractions after the maximum 3 doses. The induction-delivery interval was 12 ± 3.6 hours. Tables 3 and 4 show that majority (75.9%) of induced parturients had successful induction leading to vaginal birth, while 24.1% had failed induction resulting in emergency caesarean section for various reasons such as fetal distress, prolonged labor, cephalopelvic disproportion, and cord prolapse. Most (80.5%) of the babies delivered weighed between 2.5 and 3.9 kg, 17.5% weighed less than 2.5 kg, and 2% weighed 4 kg and above.

## 4. Discussion

The process of induction of labor requires the intervention of a skilled birth attendant to prevent undue morbidity and mortality. Despite the fact that 86.2% of parturient in this study were booked, only 40.3% had hospital delivery. It was also noted that just like in most Nigerian states and other similar resource poor settings, most of the parturient booked for antenatal care in their third trimester, thus not benefiting from some early pregnancy prophylactic interventions.

The induction rate of 11.5% in this study was much higher than the 6.6% reported from Maiduguri in Nigeria and an average of 4.4% (range of 1.4%–6.8%) reported by Bukola et al. [3, 19] but within the 9.9–33.7% in the United States [2]. The commonest indication for induction of labor which was postdate in 45.8% is similar to the 46.8% reported in Maiduguri, Nigeria [3]. Postdate and hypertensive diseases of pregnancy were both reported as the commonest indication in Sokoto by Ekele et al. [20], but this is at variance with the report by Bukola et al. and also Abdul in Zaria which identified prelabor rupture of membranes and hypertension in pregnancy as the commonest indications [19, 21]. Accurate determination of gestational age to ascertain a post date pregnancy may sometimes be an obstetric dilemma due to unsure date of the last menstrual period and nonavailability of early dating ultrasound scan as often the case in resource constrained settings. Other indications for induction in this study were term premature rupture of membranes, intrauterine fetal death, preeclampsia, preterm premature rupture of membranes, eclampsia, intrauterine growth restriction, and gestational diabetes; these indications are similar to those reported in Maiduguri, Zaria, Sokoto, as well as, India, Canada, and the United States [3, 20–24]. These indications were mainly for high risk pregnancies that required termination with early induction of labor to prevent perinatal and maternal morbidity and mortality. In such situation, the maternal and fetal risk associated with continuation of the pregnancy should be weighed against the benefits of discontinuation, especially considering the poor health seeking behavior of parturient in sub-Saharan Africa as well as the lack of modern facilities for fetomaternal surveillance.

The commonest method of induction (misoprostol) used for cervical ripening in this study could be followed by oxytocin titration after an interval of 6 hours in those in whom contractions were less than three in ten minutes. In all cases in which misoprostol was used, the vaginal route of administration was preferred, even though there have been reports of other equally effective routes such as the sublingual and rectal routes [25]. The 50-microgram dosage used was in line with the World Health Organization recommendation of 25–50 micrograms [26–28]. Although misoprostol is currently not approved for induction of labor in the United States and United Kingdom, its use is approved by several local departmental protocols in Nigeria, since the other prostaglandins such as Pg E2 recommended by the American College of Obstetricians and Gynecologists (ACOG) and the Royal College of Obstetricians and Gynecologists (RCOG) are usually unaffordable and unavailable in our resource constrained setting [29, 30]. To prevent the risk of possible uterine rupture associated with the use of the prostaglandin E1 analogue (misoprostol), intracervical extraamniotic Foleys catheter and membrane sweep are preferred methods in those at high risk of possible uterine rupture, such as the grand multiparae, those with previous uterine surgeries or uterine dilatation and curettage. The use of these methods in a reasonable proportion of our subjects was therefore not surprising since a significant proportion of the participants were multiparous with some having risk factors for uterine rupture.

The overall induction-delivery interval of 12 ± 3.6 hours in this study was similar to the 12 ± 5.2 hours reported by Abdul in Zaria [21] and comparable to an interval of 8.7 ± 2.4 hours versus 11.9 ± 2.7 hours for misoprostol and Foleys catheter, respectively, reported by Owolabi in Ile-Ife, Nigeria [31]. Also the overall successful induction rate of 75.9% in this study was similar to the 70.3% reported in the United States [32] but less than the 96% and 91% for misoprostol and Foleys catheter, respectively, reported by Ekele in Sokoto, Nigeria [20, 33]. However, the success rate of 77.7% versus 40% for misoprostol and catheter, respectively, in this study is far lower than the finding in Sokoto [20, 33], while Tabowei in Kwale, Nigeria, reported no difference in success and failure rates when misoprostol was compared with catheter [31]. Other methods such as amniotomy and oxytocin, Foleys catheter with amniotomy/oxytocin as well as membrane sweep used in this study were reportedly used in Benin City and Ile-Ife for similar indications with similar outcomes [34, 35]. Some researchers have also suggested a possible role for nonpharmacologic methods like the use of castor oil, enema, nipple stimulation, sexual intercourse, hot bath, and acupuncture, but these are yet to be subjected to randomized control trials and thus have limited evidence to support their effectiveness [2]. In Lagos, Nigeria, Ezechi reported the reasons for failed induction with misoprostol to include cephalopelvic disproportion, fetal distress, prolong labor, and antepartum hemorrhage [36]; these were similar to our findings. Most of the babies delivered by the parturient in this study were average weight babies with weight ranging mostly between 2.5 kg and 3.9 kg, suggesting that macrosomia was not a major concern for failed induction. It is important to ensure proper fetomaternal surveillance during induction because of its significant role in the safe management of parturient with high risk pregnancies and also as way of preventing perinatal and maternal morbidity and mortality that could complicate such pregnancy and the induction procedures.

## 5. Conclusion

Induction of labor is beneficial and safe in high risk pregnancies when the benefits of early delivery outweigh the risk of continuation, but this is not without attendant complications and failures which can be significantly reduced with proper patient selection, good preparation, as well as adequate fetomaternal monitoring to ensure a favorable obstetric outcome of a healthy mother and baby which are the targets of the safe motherhood initiative as well as the 4th and 5th millennium development goals.

## Figures and Tables

**Table 1:** Sociodemographic characteristics, parity and gestational age at booking (*N* = 1510).

Variables	*n*	Percentage (%)
Age (years)		
<20	97	6.4
20–24	387	25.7
25–29	705	46.7
30–34	284	18.8
35–40	35	2.3
>40	2	0.1
Educational status		
None	38	2.6
Primary	375	24.8
Secondary	455	30.1
Tertiary	642	42.5
Parity		
0	430	28.5
1–4	964	63.8
≥5	116	7.7
Gestational age at booking (weeks)		
<13	30	2.0
13–27	252	16.7
28–42	1208	80.0
>42	20	1.3

**Table 2:** Methods and indications for induction (*N* = 1510).

Variables	*n*	Percentage (%)
Method		
Misoprostol	1181	78.2
Foleys catheter	25	1.7
Foleys catheter and amniotomy/oxytocin	138	9.1
Amniotomy/oxytocin	106	7.0
Membrane sweep	12	0.8
Membrane sweep and amniotomy/oxytocin	48	3.2
Main indications		
Postdate	691	45.8
Term PROM	482	31.9
IUFD	187	12.4
Pre eclampsia	71	4.7
Preterm PROM	56	3.7
Eclampsia	11	0.7
IUGR	6	0.4
Gestational DM	6	0.4

As a departmental protocol, induction for postdate is undertaken at 41 weeks + ^3^days (40 weeks + ^10^ days) in uncomplicated pregnancies.

Diagnostic criteria for preeclampsia: hypertension in the second half

of pregnancy (≥20 weeks gestation) with blood pressure ≥140/90 mmHg taken on two occasions at least 6 hours apart in the presence of significant proteinuria (>300 mg of protein in a 24-hour urine collection or ≥2+ of protein on dip stick).

Diagnostic criteria for gestational diabetes: based on fasting blood sugar of ≥7.0 mmol/L (≥126 mg/dL) and 2-hour postprandial of ≥11.1 mmol/L (≥200 mg/dL).

IUGR: Intrauterine growth restriction.

PROM: Premature rupture of membranes.

DM: Diabetes mellitus.

**Table 3:** Outcome of induction/mode of delivery of induced parturient.

Variable	*n*	Percentage (%)
*Methods and mode of delivery *		
Misoprostol, *N* = 1181		
Vaginal delivery	918	77.7
Caesarean section	263	22.3
Foleys catheter, *N* = 25		
Vaginal delivery	10	40.0
Caesarean section	15	60.0
Foleys catheter and amniotomy/oxytocin, *N* = 138		
Vaginal delivery	108	78.3
Caesarean section	30	21.7
Amniotomy/oxytocin, *N* = 106		
Vaginal delivery	78	73.6
Caesarean section	28	25.4
Membrane sweep, *N* = 12		
Vaginal delivery	3	25
Caesarean section	9	75
Membrane sweep and amniotomy/oxytocin, *N* = 48		
Vaginal delivery	29	60.4
Caesarean section	19	39.6

**Table 4:** Reasons for failed induction (*N* = 364).

Variables	*n*	Percentage (%)
*Reasons for failed induction *		
Misoprostol, *N* = 263		
Fetal distress	103	39.2
Prolonged labor	99	37.6
Cephalopelvic disproportion	60	22.8
Cord prolapse	1	0.4
Foleys catheter, *N* = 15		
Fetal distress	2	13.3
Prolonged labor	13	86.7
Foleys catheter and amniotoy/oxytocin, *N* = 30		
Fetal distress	10	33.3
Cephalopelvic disproportion	18	60
Cord prolapse	2	6.7
Amniotomy/oxytocin, *N* = 28		
Fetal distress	5	17.8
Prolonged labor	22	78.6
Cord prolapse	1	3.6
Membrane sweep, *N* = 9		
Prolonged labor	9	100
Membrane sweep and oxytocin, *N* = 19		
Fetal distress	4	21.1
Prolonged labor	15	78.9
